# Oophorectomy for Cancer

**Published:** 1897-10-16

**Authors:** 


### OOPHORECTOMY FOR CANCER.

The very curious tendency to retrogression which has lately been observed to occur in cancerous growths after the performance of oophorectomy is worthy of the most careful consideration. It must, however, be taken along with other facts which show that a similar atrophic change may take place entirely independently of any such operation. It is quite necessary to take this larger view of the subject, as has been done by Mr. Stanley Boyd in a recent paper, if we are to estimate at all correctly the position of oophorectomy as a means of modifying the cancerous process.

After relating the cases of retrogression brought before the Clinical Society last November, and the experiences of Dr. Beatson (see The Hospital, December 5th, 1896), Mr. Stanley Boyd gives five cases of his own, in one of which the result has been very good, and in another very marked—at least for a time—and in both has been sufficient to prove that the removal of the ovaries sometimes has a distinct influence on the progress of the disease.

Not unnaturally the question has been asked whether these results have arisen in consequence of some special relationship between ovary and breast. But this would seem not to be the case, for the breast had been previously removed in each case, the nodules in question occurring in the skin and subcutaneous tissue. Clearly the matter is one of nutrition, and is to be explained, if it is to be explained at all, by the effect on the general nutritive processes which is produced by removal of the ovaries.

Without for a moment entering on the much disputed question whether or not cancer is due to the presence of a parasite, or the still further question whether, if a parasite there be, it produces its evil effects by stimulating the epithelial elements to overgrowth, or by lessening that power, whatever it is, by virtue of which they are normally kept in their proper place, we have at least the fact that cancer is an infective process, a real auto-infection, due to the intrusion of the cell elements of one system among those of another, where under altered conditions of nutrition they grow amazingly. When once implanted the progress of the disease obviously depends much on the relative nutrition, or, if we prefer another word, the relative vitality of the invaded and the invading tissues, and the influence of oophorectomy on its progress is probably but one part of the well recognised influence of the artificial production of the menopause upon general nutrition. Whether this is due to the suppression of some hypothetical "internal secretion" or of some equally hypothetical "trophic" influence springing from the ovaries, is another matter. In any case, just as we see glossy skin and other variations in the nutrition of the epithelium under various conditions, which are regarded as trophic by some and toxæmic by others (and perhaps both are right), so we see variations in the apparent malignancy of cancer. It is a perfectly common experience to find that although on the whole a case of cancer may take a downward course the rate of progress varies greatly at different times and in different parts. Occasionally it may be held in abeyance for a season, healing may take place in some parts while the disease in others is eating away the tissues, and in some rare cases, as in those brought before the Clinical Society, complete retrogression may take place. The interest which attaches to oophorectomy is that it seems to be one means, possibly one among a dozen which may yet be discovered, by which such changes of nutrition may be induced. Perhaps in the end this operation may find its real place as an aid to other surgical procedures, for it is quite clear that, in many cases in which surgery is for a time successful but recurrence ultimately takes place, a very slight nutritional change might have turned the scale.

